# Military medical research on internal diseases in modern warfare: new concepts, demands, challenges, and opportunities

**DOI:** 10.1186/s40779-021-00313-8

**Published:** 2021-03-12

**Authors:** Guang-Dong Liu, Nan Wang, Hai-Ming Wang, Xin Li, Jun-Jie Shao, Zi-Fan Liu, Min Jiang, Lin Wang, Zi-Kai Wang, Meng Li, Xue-Ying Cao, Jiang Wang, Ran Zhang, Yun-Dai Chen

**Affiliations:** 1 Division of Health Services, Chinese PLA General Hospital & Chinese PLA Medical School, Beijing, 100853 China; 2 Department of Cardiovascular Medicine, Chinese PLA General Hospital & Chinese PLA Medical School, 28 Fuxing Road, Beijing, 100853 China; 3 Department of Health Services, the First Medical Center of Chinese PLA General Hospital, Beijing, 100853 China; 4 The First Clinical Medical College of Inner Mongolia Medical University, Hohhot, 010059 China; 5 Department of Gastroenterology, the First Medical Center of Chinese PLA General Hospital, Beijing, 100853 China; 6 Department of Hematology, the First Medical Center of Chinese PLA General Hospital, Beijing, 100853 China; 7 Department of Nephrology, the First Medical Center of Chinese PLA General Hospital, Beijing, 100853 China; 8 Department of Respiratory and Intensive Medicine, Chinese PLA General Hospital, Beijing, 100853 China

**Keywords:** Military medicine, Internal diseases, Modern battlefield

## Abstract

Battlefield internal medicine aims at the treatment of combatants and noncombatants with various internal diseases on the battlefield. The military medical research on battlefield internal diseases focuses on the pathogenesis, clinical management, and prevention of internal diseases under military war conditions. In both wartime and peacetime, the soldiers suffer from more internal diseases than surgical wounds. With the introduction of high-tech weapons, including chemical, physical, and biological agents, a large number of special internal illnesses and casualties will appear in future wars. The battles often occur in special environments, such as high or low temperatures, plateau or polar areas, and micro- or hyper-gravity. The current theories of battlefield internal medicine are mainly derived from wars decades ago and cannot meet the needs of military medical support under the conditions of modern warfare. Therefore, the military medical research on battlefield internal medicine should be based on contemporary military situations, focus on the purpose of treating battlefield internal diseases, and adhere to the actual needs of the troops in peacetime and wartime. We should investigate the pathogenesis of battlefield internal diseases and explore the threats that may arise in future wars to ensure the advancement of battlefield internal medicine. This review highlights new concepts, demands, challenges, and opportunities for the further development of military medical research on battlefield internal medicine.

## Background

Military medicine focuses on the art and science of medicine that is practiced in a military context with the primary goal of contributing to the success of the military mission. Military medicine constitutes the specialties of both general practice and occupational medicine under the conditions of military activities and strives to establish an effective health care system during wars.

A study enrolling 56,763 American casualties injured during the Afghanistan and Iraq wars (2001–2017) showed that the casualty rate decreased from 20% in the early Afghanistan war to 8.6% in 2018, and the casualty rate in the Iraq war fell from 20.4 to 10.1% [1]. The survival rate of the worst casualties more than tripled over a 16-year period. These improvement benefits were derived, at least in part, from the significant developments of military medical research, and also reflects the historical trend of military medical progress during major conflicts. During World War II, the Soviet army medical and health system first recognized the importance of internal disease treatment under war conditions and proposed battlefield internal medicine as one of the main branches of military medicine [2]. During the Korean War and the counterattack against Vietnam, the Chinese army also concluded that it was necessary to organize the battlefield internal medical system [3]. The theoretical basis of battlefield internal medicine at this stage is mainly derived from wars decades ago, but the theory of battlefield internal medicine in modern warfare has not been effectively developed.

Here we highlight the new concepts and demands of modern warfare for military medical research on battlefield internal medicine and address the opportunities and challenges in this field.

## New concepts of military medical research on battlefield internal medicine

Battlefield internal medicine is an open and sustainable military medical discipline that keeps up with the urgent needs of modern warfare and continuously adapts to the complex situation of military warfare. From the Gulf War, the Second Chechnya War, and the Iraq War, modern warfare is gradually infused with high-tech factors and the lethality of the updated weapons is constantly increasing. In the early stage of trauma, the injured patients are in an unstable stage and internal diseases are prone to complications, which will delay recovery from trauma [4]. The multiple organ injury and posttraumatic stress disorder is common after trauma on the battlefield [5, 6]. During World War II, cardiovascular shock was the major organ system that limited survival after injury [7]. In the Second Chechnya War, approximately 40% of the severely injured surgical patients had post-traumatic acute kidney injury and nearly one-third died of respiratory failure [8]. Modern high-tech weapons with much greater lethality, such as nuclear weapons, lasers, and microwaves, have developed rapidly, which leads to radiation injuries, gene mutations, or organ damage. The severe cold plateaus, coastal hot climates, and weightlessness significantly increase the incidence of internal diseases [9]. After analysis of seven lunar astronauts, 43% of astronauts died of cardiovascular disease, which is higher than that of healthy people of the same age in a low orbit [10]. During the recent conflicts in Iraq and Afghanistan, the most common non-communicable diseases requiring hospital admission were gastrointestinal, joint, mental, and post-traumatic stress disorders [11]. Army soldiers living in the dry zone of Sri Lanka were faced with the risk of methicillin-resistant *Staphylococcus aureus* infection [12]. Therefore, modern military medical research on battlefield internal medicine will inevitably face much more complex conditions of communicable and non-communicable diseases.

Modern high-tech weapons and the transformation of the battlefield dimension under modern warfare expand the concepts of battlefield internal medicine. Therefore, the concepts of military medical research on battlefield internal medicine should be integrated with new elements, including not only internal diseases related to traditional weapons or the battlefield, but new-concept weapons or new battlefields in modern warfare. The new content will not only focus on the pathogenesis and new strategies for battlefield internal diseases, but professional training and education in this field.

## Demands for military medical research on battlefield internal medicine

Modern battlefield internal medicine has exceeded the scope of common internal diseases under war conditions and continuously extended to epidemiology, pathogenesis, new weapon damage, and military hygiene. These changes raise new demands for military medical research on battlefield internal medicine.

### New demands for research on internal diseases caused by modern military missions

In several other civil or world wars, the incidence of internal diseases increased significantly and the traditional battlefield surgery, which mainly treats trauma, can no longer meet the requirements of military medicine in modern warfare. In classic foreign battles, non-combat downsizing caused by internal diseases accounted for approximately 20% [13]. An editorial in the *British Medical Journal* pointed out that gastrointestinal diseases are important causes of disability in the British Expeditionary Force [14]. During the Iraq War (1992–1997), norovirus outbreaks and sporadic diseases were very common among ground forces and were important causes of acute gastroenteritis and loss of routine duties [15]. The mortality rate of infectious diseases was 8% (153,000 total injuries) in World War I, 4.5% (599,724 total injuries) in World War II, 2.5% (77,788 total injuries) in the Korean War, and 3.6% (96,811 total injuries) in the Vietnam War, 2.1% (143 total injuries) in Desert Storm (7th Corps), and 6.4% (62 total injuries) in Somalia [16].

Respiratory diseases are also the main downsizing factor, and acute and chronic pneumonia are also common diseases in armies. After the Korean War (1950–1953), nearly 6.5% of the population in South Korea had active tuberculosis. The mortality rate for tuberculosis in 1942 and 1954 was 71.7/100,000 and (300–400)/100,000, respectively, which strongly indicated that wars were a major cause of the tuberculosis epidemic in South Korea [17]. Acute kidney injury caused by burns in the Iraq and Afghanistan wars was common among military combatants, with a prevalence of 23.8 and 29.9%, respectively [18]. Among 3807 US soldiers who were injured during the Iraq and Afghanistan wars (2002–2011), the incidence of acute renal failure was 12.5% [19]. According to a US Health and Nutrition Survey, including 263,430 soldiers and 4997 civilians, the ideal cardiovascular health compliance rate in soldiers was lower than that of civilians (especially in males) [20]. The trauma was associated with subclinical atherosclerosis in young adults following the Bosnia and Herzegovina wars (1990–1995) with higher levels of elevated triglycerides and carotid intima-media thickness than non-trauma-exposed subjects [21]. According to the data from 1960 to 2000 in > 100 countries, all types of armed conflict will increase the cardiovascular mortality of males and females because the exhaustion of healthcare resources and the general interruption in daily life results in excessive stress and burden [22]. From the abovementioned findings, it can be seen that wartime internal diseases are significantly different from non-wartime internal diseases and have different characteristics in the pathogenesis, clinical symptoms, morbidity, and course of diseases. Therefore, the establishment of systemic basic and military medical research on battlefield internal medicine is of great significance for reducing the non-combat downsizing of military combatants and improving combat capabilities.

### New demands for research on internal diseases caused by special wartime environment

The wartime geographic environment also pushes the development of battlefield internal medicine. Chinese borders are mostly high mountains or coastal areas. At the Tibet border, the average altitude is > 3000 m, the low air pressure is severe, the lack of oxygen, the natural environment, and the climate are bad, and the transportation is underdeveloped. The concomitant diseases of the respiratory, circulatory, nervous, and digestive systems on the plateau are the main reasons for the non-combat downsizing of troops [23]. The battlefield internal medicine under tropical conditions also needs to strengthen the prevention of diseases, such as insect bites, chemical poisoning, and heat shock [24]. The medical support in the desert and aerospace fields is also an important driving force for our efforts to develop military medical research on battlefield internal medicine [25].

### New demands for research on internal diseases caused by high-tech weapons

With the continuous introduction of high-tech weapons, including chemistry, physics, and biology, a large number of special internal illnesses and casualties will appear in future wars. Due to severe toxicity and multiple poisoning ways of chemical weapons, the prevention is often difficult and the mortality rate of soldiers attacked by chemical weapons is generally high. In the case of radar exposure, microwaves, and nuclear strikes, the radiation injury is a common type of injury in modern warfare, often leading to brain injury and an increased risk of cancer with mortality rate (MR = 0.81, 95% CI: 0.78–0.83) and relative risk (RR = 0.87, 95% CI: 0.75–0.99) [26, 27]. Biological warfare agents are new types of weapons that have emerged in modern warfare, which are based on pathogenic microorganisms or the synthesis of new types through genetic methods with rapid infection, severe illness, a long course of disease, and a high mortality rate [28]. Although the surgical damage is less severe, human energy metabolism, neurocognition and other functions, and severe internal disorders are affected. Therefore, traditional battlefield internal medicine that focuses on internal disorders cannot deal with medical support in the settings of modern high-tech warfare. New concept weapons and new combat environments urgently need to meet new demands for internal medicine.

## Challenges and opportunities for military medical research on battlefield internal medicine

The demands for military medical research on battlefield internal medicine in modern warfare are constantly expanding, which promotes new challenges and opportunities in this field.

### Challenges and opportunities of establishing and improving the education system in troops

Internal diseases do not receive sufficient attention and the troops do not receive adequate education, and there is a lack of complete, systematic, and standardized documents. Healthcare experience and participation in a training program will improve the level of knowledge [29]. We are now faced with opportunities to re-establish the education system of military medicine and establish the imperative for military-oriented medical research. Military medical research needs an independent educational system because only specialized, professional, and in-depth research can promote the development of battlefield internal medicine. The Armed Forces need to rely on the rich resources of universities and institutes to deal with current issues in modern warfare era. Both Chinese and Western Armies have reached a consensus that military-civilian integration will complete the transition from simple military hygiene into overall well-being [30].

### Challenges and opportunities of improving the theory and practice system of medical support

Military internal medicine constitutes both general practice and special characteristics of military settings, which should not be separated. Military medical research should focus on improving the theory and practice system. Military medical staff need to build a more scientific theoretical knowledge and practice system of battlefield internal medicine focusing on the actual deployment of troops and the requirements of the future information warfare for the quality and knowledge of medical support staff. Therefore, the military medical research should base on the contemporary military situations, and stick to the actual needs of the troops in peacetime and wartime. We should integrate physical, biological, psychological, social, and other factors to modern battlefield internal medicine under war conditions. For instance, recruiting young people who are physically and mentally healthy is very important. In-depth and extensive battlefield medical research will help to identify who is susceptible to battlefield internal diseases in modern warfare. The organization form of battlefield internal medicine should be continuously studied, including the prediction of various internal diseases, the reduction of downsizing from internal diseases, and the evacuation of the wounded to rear battle. We should face future military medical research and explore the threats that may arise in future wars to ensure the advancement of battlefield internal medicine.

### Challenges and opportunities of innovative basic research to improve clinical practice strategies

The pathogenesis and management of battlefield internal diseases are challenging. Military medical research should focus on the basic mechanism to better understand the pathogenesis of battlefield internal diseases and develop clinical practice strategies. For instance, our ground-based studies on the molecular mechanism of cardiovascular dysfunction in the settings of microgravity benefit developing preventive strategies of postflight cardiovascular deconditioning in astronauts [25, 31, 32]. The latest proposal to use stem cells in the treatment of battlefield injuries is very promising. This treatment technology could not only treat the common damage of various organ systems, but is also expected to improve the treatment effect of radiation injury and other damage caused by new concept weapons [33]. Keeping up with the new research on the pathogenesis of sepsis and multiple organ dysfunction syndrome with a high mortality rate will provide future battlefield internal medicine with a new therapeutic strategy that is more suitable for warfare conditions [5]. Uncertainties under special battlefields still exist. The venous thromboembolism during microgravity exposure has been reported; however, the underlying mechanism remains to be established [34]. Finally, military medical research on battlefield internal medicine should promote evidence-based guidelines on the management of battlefield injuries.

## Conclusion

The demands for military medical research on battlefield medicine in the future modern warfare is constantly expanding, and the battlefield medicine is far beyond the scope of traditional trauma. To better guarantee the victory of modern warfare and to increase the combat effectiveness of the troops more scientifically, strengthening the development of military medical research on battlefield internal medicine is an imperative military medical task.

## Data Availability

The data and materials are all available from this review.
